# Clinical Emergence and Genomic Characterization of Aztreonam–Avibactam-Resistant *Escherichia coli* ST410 Isolates in China

**DOI:** 10.3390/antibiotics15080750

**Published:** 2026-08-03

**Authors:** Xiaojie Li, Pu Li, Junchao Feng, Zhaoyang Fang, Sheng Liu, Cheng Guo, Bo Hu

**Affiliations:** 1 Department of Laboratory Medicine, The Third Affiliated Hospital of Sun Yat-Sen University, Guangzhou 510630, China; lixj55@mail.sysu.edu.cn (X.L.);; 2 School of Public Health, Sun Yat-Sen University, Guangzhou 510080, China; 3 Nanning Center for Disease Control and Prevention, Nanning 530023, China; 4 National Medical Products Administration Key Laboratory for Quality Monitoring and Evaluation of Vaccines and Biological Products, Guangzhou 510080, China

**Keywords:** aztreonam–avibactam resistance, *Escherichia coli*, PBP3 mutations, ST410

## Abstract

Background: Aztreonam–avibactam (ATM-AVI) is an important therapeutic option for infections caused by metallo-β-lactamase-producing Enterobacterales. However, the emergence of resistance may limit its clinical effectiveness. Here, we report two high-level ATM-AVI-resistant *Escherichia coli* isolates recovered from critically ill patients in a tertiary hospital in Guangzhou, both of whom had prior exposure to broad-spectrum antimicrobial agents. Methods: Antimicrobial susceptibility testing was performed by broth microdilution. Whole-genome sequencing (WGS) was conducted using the Illumina NovaSeq and Oxford Nanopore Technologies platforms for hybrid assembly. Resistome analysis, multilocus sequence typing (MLST), plasmid replicon typing, and phylogenomic analysis were performed. Comparative genomics with global ST410 isolates was used to investigate the evolutionary origin. Results: Antimicrobial susceptibility testing revealed high-level resistance to ATM-AVI. Hybrid whole-genome sequencing showed that both isolates belonged to the globally disseminated high-risk ST410 lineage and carried *bla*_NDM-5_, CTX-M-type extended-spectrum β-lactamase genes, and an identical four-amino-acid insertion in penicillin-binding protein 3 (*ftsI*_I334IYRIK). Both isolates exhibited highly conserved chromosomal backbones, differed by only 29 core-genome single-nucleotide polymorphisms, and clustered within a China-associated ST410 clade. Notably, their key resistance determinants were located on distinct plasmid backgrounds: *bla*_NDM-5_ was found on a predicted conjugative IncFIB/IncFIC plasmid in one isolate and on an IncX1 element with potential mobilization in the other. Conclusions: These findings provide important clinical evidence of high-level ATM-AVI resistance in *Escherichia coli* ST410 isolates in China and highlight the emergence of ATM-AVI resistance-associated determinants within a high-risk genomic background. Active phenotypic and genomic surveillance is warranted as ATM-AVI enters broader clinical use.

## 1. Introduction

Carbapenem-resistant Enterobacterales (CRE) have become a major global public health threat because of their rapid dissemination, limited therapeutic options, and association with high morbidity and mortality [1]. Among the mechanisms underlying carbapenem resistance, metallo-β-lactamases (MBLs), particularly New Delhi metallo-β-lactamase (NDM), are of particular concern. These enzymes hydrolyze nearly all β-lactams and are not inhibited by currently available serine β-lactamase inhibitors. NDM-producing Enterobacterales also frequently co-harbor extended-spectrum β-lactamases (ESBLs), AmpC β-lactamases, and additional resistance determinants, further restricting treatment options [2].

Aztreonam–avibactam (ATM-AVI) has emerged as a promising therapeutic strategy for infections caused by MBL-producing Enterobacterales [3]. Aztreonam is intrinsically stable against hydrolysis by MBLs, whereas avibactam inhibits many co-produced serine β-lactamases, including ESBLs, AmpC enzymes, and some class D β-lactamases, which would otherwise compromise aztreonam activity [4]. The combination may therefore restore activity against isolates producing NDM or other MBLs in the presence of additional β-lactamases [5]. In August 2025, ATM-AVI was first introduced in China for adult complicated intra-abdominal infections and hospital-acquired pneumonia, including ventilator-associated pneumonia caused by Gram-negative pathogens with limited treatment options, representing an important advance for difficult-to-treat Gram-negative infections.

However, the clinical value of ATM-AVI may be undermined by both pre-existing and treatment-emergent resistance. Recent surveillance studies have identified baseline ATM-AVI resistance among MBL-producing Enterobacterales, with higher frequencies in specific sequence types, particularly *Escherichia coli* (*E. coli*) ST361 and ST167 [6]. Several mechanisms have been implicated in reduced susceptibility or resistance to ATM-AVI, including alterations in penicillin-binding protein 3 (PBP3), co-production or overexpression of β-lactamases with enhanced aztreonam-hydrolyzing activity, and changes in membrane permeability or efflux activity [7,8]. Among these, alterations in *ftsI*, which encodes PBP3, are among the most frequently reported mechanisms. PBP3 is a primary target of aztreonam, and four-amino-acid insertions in its transpeptidase domain, such as YRIN- or YRIK-like insertions, can reduce the binding affinity of aztreonam [7,9,10]. These mechanisms are clinically important because treatment options for infections caused by MBL-producing and ATM-AVI-resistant organisms remain limited.

*E. coli* ST410 is a globally disseminated high-risk clone that is increasingly associated with multidrug resistance, carbapenemase production, and acquisition of mobile resistance elements, including *bla*_NDM-5_. The convergence of this high-risk clonal background with PBP3 alterations and transferable resistance plasmids may facilitate the emergence and dissemination of ATM-AVI resistance [11]. Nevertheless, the clinical occurrence and genomic context of high-level ATM-AVI resistance in *E. coli* ST410 isolates from China remain poorly characterized.

Here, we report the clinical identification and genomic characterization of high-level ATM-AVI-resistant *E. coli* ST410 isolates in China. Using antimicrobial susceptibility testing, hybrid whole-genome sequencing, plasmid analysis, and comparative phylogenomics, we characterized their resistance determinants and placed them within the globally disseminated ST410 lineage. These findings highlight the emergence of ATM-AVI resistance-associated determinants in a high-risk *E. coli* genomic background and support the need for active phenotypic and genomic surveillance as ATM-AVI becomes more widely used.

## 2. Results

### 2.1. Clinical Presentation and Microbiological Characteristics

*E. coli* ZSSY10862 was recovered from a cerebrospinal fluid (CSF) sample from a 30-year-old man with primary central nervous system diffuse large B-cell lymphoma (PCNS-DLBCL), hydrocephalus, and interstitial cerebral edema. The patient underwent CSF drainage followed by right ventriculoperitoneal shunt placement in September 2025. During hospitalization, he developed a secondary central nervous system infection, severe pneumonia, and a complicated urinary tract infection. He presented with fever and tachycardia, accompanied by leukocytosis (19.78 × 10^9^/L; neutrophils, 88.2%) and an elevated procalcitonin (0.547 ng/mL). CSF cultures yielded Gram-negative rods within 12 h, and the isolate exhibited a multidrug-resistant phenotype.

A second isolate, *E. coli* ZSSY9436, was recovered from the sputum of a patient with recurrent polymicrobial pneumonia and multiple comorbidities, including cardiac insufficiency, hypertension, chronic kidney disease, Alzheimer’s disease, and post-stroke sequelae. Between September 2024 and February 2026, the patient was hospitalized nine times for severe pneumonia and required intubation on several occasions. Chest radiographs showed bilateral pulmonary infiltrates. Laboratory findings revealed leukocytosis (13.12 × 10^9^/L; neutrophils, 79.7%) and elevated procalcitonin (0.426 ng/mL). The patient received multiple courses of broad-spectrum antimicrobial therapy during hospitalization and was discharged afebrile on 4 March 2026, with clinical symptoms resolved. The sputum isolate also exhibited a multidrug-resistant phenotype.

**Figure 1:**
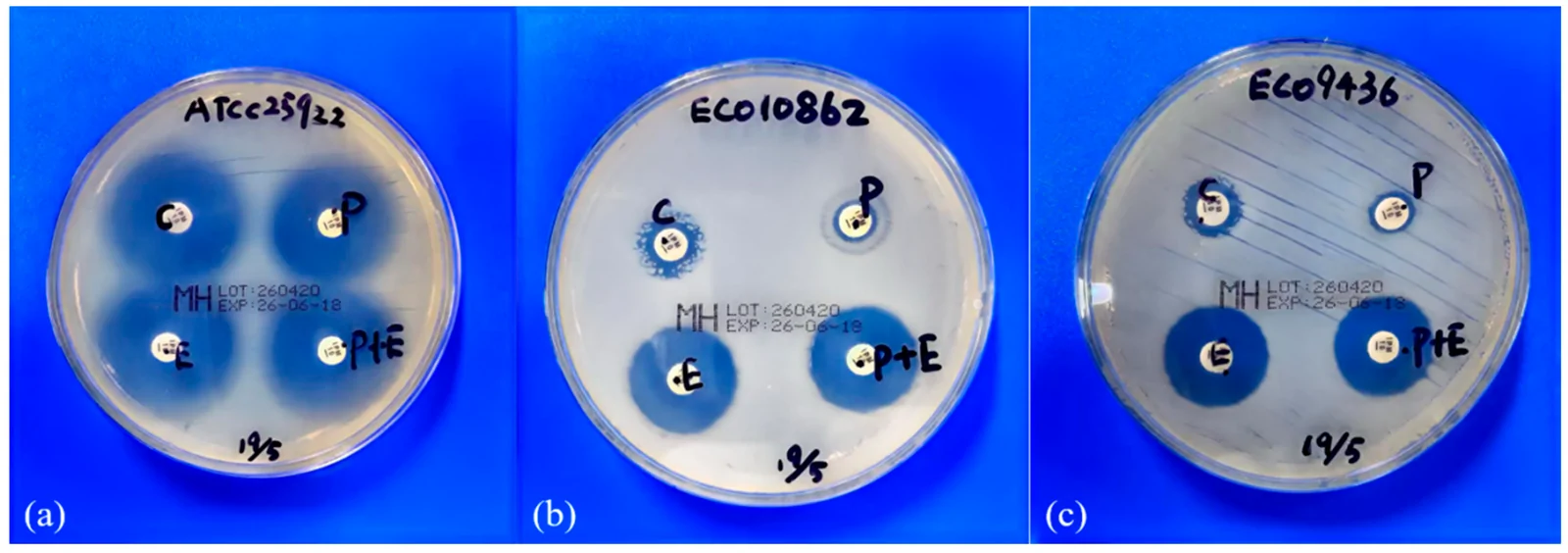
Carbapenemase phenotype results for *E. coli* ZSSY10862 (**b**) and *E. coli* ZSSY9436 (**c**). The disk Diffusion Assay illustrates the contrast in inhibition zones between clinical isolates and the susceptible control strain *E. coli* ATCC 25922 (**a**). C: IMP control; P: IMP + 5 µL ABP (3-Aminobenzeneboronic acid); E: IMP + 5 µL Na_2_EDTA (Ethylenediaminetetraacetic acid disodium salt); P + E: IMP + 5 µL ABP + 5 µL Na_2_EDTA.

Antimicrobial susceptibility testing showed that both *E. coli* ZSSY10862 and ZSSY9436 were resistant to all β-lactams tested, including carbapenems, ceftazidime-avibactam, and aztreonam–avibactam (Table 1). Both isolates exhibited high-level resistance to carbapenems (MIC ≥ 16 µg/mL) and to aztreonam–avibactam (MIC = 32 µg/mL). Ceftazidime-avibactam resistance was also observed, with an inhibition zone diameter of 10 mm. Carbapenemase production was confirmed by a modified carbapenemase inhibition test, interpreted as positive when a clover leaf-like indentation of *E. coli* ATCC 25922 was observed within the inhibition zone. The combined disk method for carbapenemase classification showed that an increase in zone diameter of ≥4 mm was observed with Na_2_EDTA (MBL inhibitor), indicating that both isolates were MBL-positive. The carbapenemase inactivation test further indicated that both isolates likely produced class B metallo-β-lactamases (Figure 1).

### 2.2. Genome Assembly, Plasmid Profiles, and Resistance Determinants of Both ST410 E. coli Isolates

**Figure 2:**
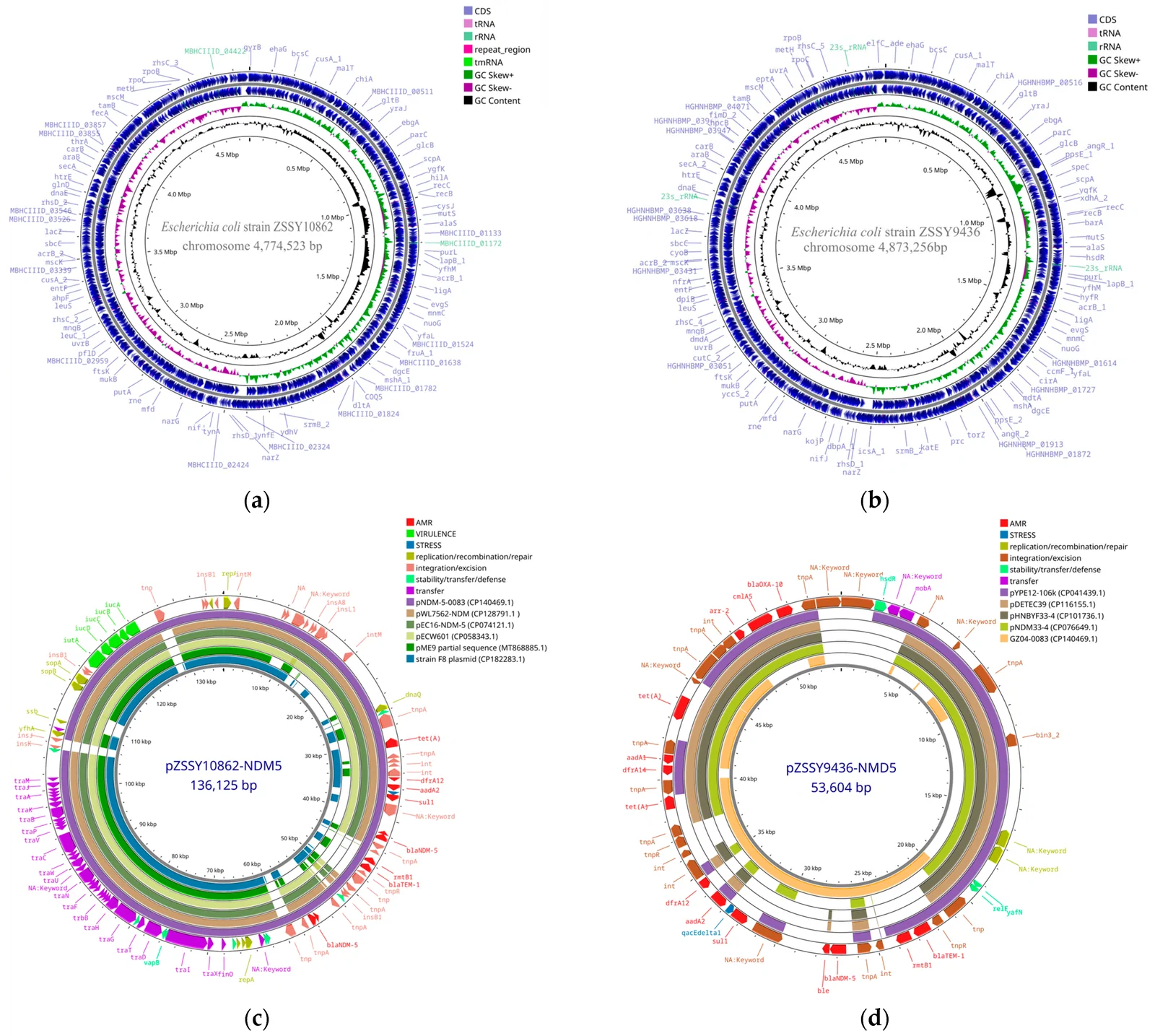
Circular genome maps of *E. coli* ZSSY10862 (**a**) and *E. coli* ZSSY9436 (**b**). (**c**) Plasmid pZSSY10862-NDM5 from *E. coli* ZSSY10862. (**d**) Plasmid pZSSY9436-NDM5 from *E. coli* ZSSY9436.

Hybrid assembly resolved *E. coli* ZSSY10862 into one circular chromosome with seven circular plasmids, and *E. coli* ZSSY9436 into a circular chromosome with nine plasmids. Both isolates were assigned to ST410 and shared several plasmid replicon types, including IncY, IncI2, IncX-type, and Col-type replicons (Table 2; Figure 2).

Both isolates carried multidrug resistance determinants centered on *bla*_NDM-5_ and CTX-M-type ESBL genes. *E. coli* ZSSY10862 harbored two copies of *bla*_NDM-5_ and *bla*_CTX-M-199_, whereas *E. coli* ZSSY9436 carried *bla*_NDM-5_ together with *bla*_CTX-M-137_, *bla*_CTX-M-14_, *bla*_TEM-1_, and *bla*_OXA-10_. Additional resistance genes associated with aminoglycosides, sulfonamides, trimethoprim, tetracyclines, and bleomycin were detected in both isolates, while rifamycin and chloramphenicol resistance determinants were detected only in *E. coli* ZSSY9436 (Table 2).

To characterize the genomic differences between *E. coli* ZSSY10862 and *E. coli* ZSSY9436, we performed whole-genome similarity and chromosome synteny analyses. The chromosomes of both isolates were highly conserved, with an average nucleotide identity (ANI) of 100.00% between *E. coli* ZSSY10862 and *E. coli* ZSSY9436. *E. coli* ZSSY10862 chromosome was fully covered by *E. coli* ZSSY9436 chromosome, whereas the alignment covered 98.05% of the *E. coli* ZSSY9436 chromosome, consistent with the larger chromosome size of *E. coli* ZSSY9436 compared with *E. coli* ZSSY10862. MUMmer-based synteny analysis further showed that both chromosomes were largely collinear, with no major chromosomal inversion or large-scale rearrangement. The major difference was localized to an approximately 92.7 kb region in *E. coli* ZSSY9436 at coordinates 819,093–911,754, which was absent from the corresponding locus in *E. coli* ZSSY10862. ICEberg3 annotation identified this region as a trnF/tRNA-Phe(gaa)-associated T4SS-type integrative and conjugative element, indicating that acquisition of this ICE represents the major strain-specific chromosomal expansion in *E. coli* ZSSY9436.

MBL genes were located on different plasmid backbones in the two isolates. In *E. coli* ZSSY10862, two copies of *bla*_NDM-5_ were located on a 136,125 bp IncFIB/IncFIC plasmid together with *ble*, *rmtB*, and *bla*_TEM-1B_, while *bla*_CTX-M-199_ was located on a 63,260-bp IncI2 plasmid. In *E. coli* ZSSY9436, *bla*_NDM-5_ was located on a 53,604-bp IncX1 plasmid within a multidrug resistance region containing *ble*, *bla*_TEM-1_, *rmtB*, *aadA*, *dfrA*, *tet(A)*, and *bla*_OXA-10_. The CTX-M-family genes in *E. coli* ZSSY9436 were mapped to an IncFIA/IncFIC plasmid and an IncI2 plasmid, respectively.

Both isolates also carried the same PBP3 alteration annotated as *ftsI*_I334IYRIK, and identical quinolone resistance-associated substitutions in *gyrA* and *parC*. These results indicated that both ST410 isolates shared several major resistance determinants but differed in *bla*_NDM-5_ copy number, CTX-M subtype, plasmid location, and accessory resistance gene content.

### 2.3. Plasmid Context and Predicted Mobility of bla_NDM-5_-Bearing Elements

To investigate the genetic context and potential mobility of the *bla*_NDM-5_ in both ATM-AVI-resistant *E. coli* isolates, contigs carrying *bla*_NDM-5_ were analyzed using MOB-suite (Table 3). In *E. coli* ZSSY10862, *bla*_NDM-5_ was located on contig 2, which carries IncFIB and IncFIC replicons and harbors relaxase genes (MOBF, MOBP), an oriT site, and an MPF_F mating-pair formation system. This plasmid was therefore predicted to be conjugative, indicating that it possesses the genetic modules required for self-transfer between bacteria.

In *E. coli* ZSSY9436, *bla*_NDM-5_ was initially identified on contig 5, which represents an incomplete, non-conjugative plasmid carrying a MOBQ relaxase but lacking both an MPF system and an oriT site (Table 3). Because contigs 3 and 5 were assigned to the same MOB-suite primary cluster, we further considered them as a hypothesized composite plasmid structure that may act cooperatively to enable conjugative transfer. The combined assembly contains MPF_F and oriT modules and is therefore predicted to be conjugative. This indicates that *bla*_NDM-5_ in *E. coli* ZSSY9436 could potentially be mobilized through co-resident plasmids or a composite plasmid structure, highlighting a possible route of horizontal transfer.

### 2.4. Molecular Epidemiology and Phylogenetic Analysis

**Figure 3:**
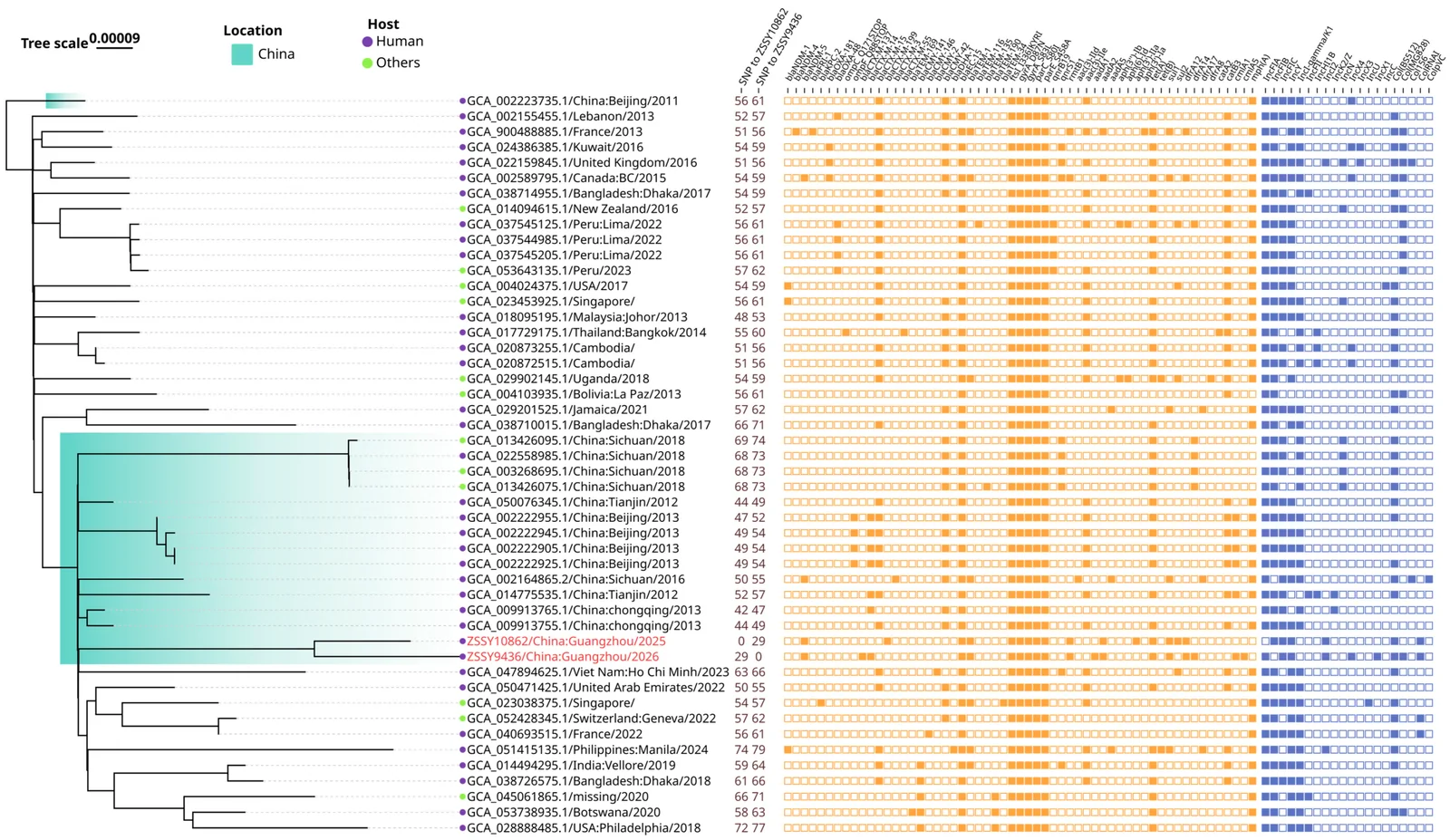
Global phylogenetic context of ATM-AVI-resistant *Escherichia coli* ST410 isolates.

To investigate the genetic relationship between the newly sequenced isolates and publicly available global ST410 genomes, a core-genome phylogenetic tree was constructed (Figure 3). *E. coli* ZSSY10862 and *E. coli* ZSSY9436 clustered within a clade composed of Chinese isolates collected between 2012 and 2018. Both isolates carried key resistance determinants associated with the ATM-AVI-resistant phenotype, including *bla*_NDM-5_ and the PBP3 insertion *ftsI*_I334IYRIK. Pairwise single-nucleotide polymorphism (SNP) analysis further supported their close genetic relatedness. The genetic difference between *E. coli* ZSSY10862 and *E. coli* ZSSY9436 was low, with a difference of only 29 core SNPs.

## 3. Discussion

This report describes two clinical isolates, *E. coli* ZSSY10862 and *E. coli* ZSSY9436, exhibiting high-level resistance to the aztreonam–avibactam combination through a complex mechanism involving NDM-5 production, a PBP3 alteration, and diverse CTX-M-type ESBLs. Both isolates belonged to the global high-risk clone ST410, an emerging lineage increasingly associated with ATM-AVI resistance worldwide.

The emergence of resistance in two patients with healthcare-associated infections, despite no prior exposure to ATM-AVI, indicates that resistant clones are already circulating in healthcare environments and have likely been selected by previous β-lactam exposure. According to the medication history of these cases, both patients were extensively treated with meropenem and piperacillin-tazobactam before the isolation of the resistant strains. We hypothesize that this antecedent antimicrobial therapy may have provided the selective pressure that enriched pre-existing resistant subpopulations. Because β-lactam antibiotics such as meropenem also target PBP3, mutations that reduce PBP3 binding affinity not only decrease bacterial susceptibility to these earlier agents but also confer a survival advantage on the mutant subpopulation, thereby driving cross-resistance to ATM-AVI. This resistance phenomenon poses serious challenges for clinical management. In the face of MBL-producing, ATM-AVI-resistant *E. coli*, available therapeutic options are exceedingly limited, leaving clinicians to rely on polymyxins, tigecycline, fosfomycin, and cefiderocol as salvage therapies [3,12,13].

Recent surveillance data from the European Centre for Disease Prevention and Control (ECDC) identified ST167 and ST361 as the primary sequence types associated with pre-treatment ATM-AVI resistance among NDM producers [9]. Our phylogenetic analysis confirms that ATM-AVI-resistant ST410 strains form a distinct clade, suggesting clonal expansion rather than independent acquisition of resistance in diverse lineages. Phylogenetic analysis further contextualizes the emergence of ATM-AVI resistance in these two isolates. Both *E. coli* ZSSY10862 and *E. coli* ZSSY9436 clustered within a China-associated ST410 clade, indicating that the resistance phenotype emerged in a high-risk *E. coli* lineage rather than in unrelated sporadic genomic backgrounds. This observation is clinically important because ST410 has been recognized as a successful multidrug-resistant lineage capable of acquiring carbapenemase genes and diverse plasmid backbones. In parallel, MOB-suite analysis showed that *bla*_NDM-5_-bearing elements in these isolates carried different conjugation-associated modules. The *bla*_NDM-5_ plasmid in *E. coli* ZSSY10862 was predicted to be conjugative, whereas the *bla*_NDM-5_-bearing IncX1 element in *E. coli* ZSSY9436 appeared to require co-resident or composite conjugative modules for mobilization. The spread of this resistance background may involve both ST410 clonal expansion and plasmid-mediated horizontal transfer. However, we acknowledge that in silico prediction of plasmid transferability has inherent limitations and follow-up conjugation experiments are required to experimentally validate the transferability of these plasmids.

The ATM-AVI combination exploits the stability of aztreonam against MBLs, while avibactam concurrently inhibits co-expressed serine β-lactamases, including SHV, TEM, CTX-M, AmpC, KPC, and OXA-48-like enzymes. Mechanistically, aztreonam itself is relatively stable against MBLs. Therefore, the presence of *bla*_NDM-5_ alone was insufficient to cause high-level ATM-AVI resistance. Previous studies have shown that MBLs have limited hydrolytic activity against aztreonam [14,15]. The activity of ATM-AVI primarily relies on avibactam inhibiting co-existing ESBLs or AmpC, thereby protecting aztreonam from hydrolysis. Recent surveillance data have outlined emerging ATM/AVI resistance mechanisms in *E. coli* clinical isolates, encompassing (i) acquired mutations in PBP3 that diminish aztreonam target affinity, (ii) reduced outer membrane permeability, (iii) efflux pump upregulation, and (iv) the emergence of β-lactamase variants capable of hydrolyzing aztreonam while remaining refractory to avibactam inhibition. The *ftsI*_I334IYRIK mutation shared by both strains is likely the key target-site basis for their reduced ATM-AVI susceptibility. PBP3 is a crucial action target of aztreonam. Studies have demonstrated that four-amino-acid insertions, including YRIK and others (YRIN, YRIP or TIPY), near position 333 of PBP3 can reduce the binding affinity between aztreonam and PBP3, and thereby increase the MICs of ATM or ATM-AVI [16,17]. However, a standalone PBP3 insertion is usually insufficient to cause full resistance. Instead, it acts as a target-modifying factor that further amplifies the resistance level in the presence of a β-lactamase background [18,19].

In this study, *E. coli* ZSSY10862 carries two copies of *bla*_NDM-5_ and *bla*_CTX-M-199_, where *bla*_NDM-5_ is located on an IncFIB/IncFIC plasmid and *bla*_CTX-M-199_ is located on an IncI2 plasmid. CTX-M-199 is a hybrid enzyme related to CTX-M-15 and CTX-M-14. Previous studies have shown that it possesses strong aztreonam-hydrolyzing capability and can confer ATM-AVI resistance on a PBP3-YRIK background [9]. In contrast, *E. coli* ZSSY9436 carries only a single copy of *bla*_NDM-5_ but concurrently possesses multiple β-lactamase genes such as *bla*_CTX-M-137_, *bla*_CTX-M-14_, *bla*_TEM-1_, and *bla*_OXA-10_. Within this strain, *bla*_NDM-5_, *bla*_TEM-1_, and *bla*_OXA-10_ are located in the multidrug resistance region of an IncX1 plasmid, while the CTX-M family genes are distributed on IncFIA/IncFIC and IncI2 plasmids, respectively. This difference indicates that under the same PBP3 insertion and NDM-5 background, different combinations of ESBLs or narrow-spectrum β-lactamases can generate sufficient aztreonam-hydrolyzing pressure, preventing avibactam from fully restoring aztreonam activity. While *AmpC* upregulation via promoter mutations has been proposed as a way to hydrolyze aztreonam when PBP3 affinity is reduced, we did not observe this mechanism in our isolates [7]. Similarly, no porin mutations (ompC/ompF) were identified in these isolates, in contrast to other ATM-AVI-resistant strains in which porin loss contributed to resistance [6]. Together, these observations suggest that ATM-AVI resistance can emerge through multiple evolutionary pathways.

Current CLSI and EUCAST guidance supports testing ATM-AVI as a fixed combination for MBL-producing isolates [20]. However, many routine clinical laboratories, particularly in resource-limited settings in China, do not yet have access to dedicated ATM-AVI susceptibility testing panels. In addition, the results of aztreonam monotherapy testing cannot reliably predict susceptibility to the ATM-AVI combination. These limitations highlight the need to strengthen laboratory detection and surveillance strategies. Where available, ATM-AVI susceptibility testing should be considered for MBL-producing Enterobacterales, especially for isolates recovered from high-risk patients such as transplant recipients and patients in intensive care units. Moreover, genomic surveillance should include screening for known *ftsI* alterations, particularly in high-risk clones such as ST410. Closer integration of phenotypic testing with genomic monitoring may help detect ATM-AVI-resistant lineages before they disseminate more widely.

In conclusion, this study provides the first clinical documentation of high-level ATM-AVI resistance in *E. coli* ST410 from China, a finding of considerable clinical concern that underscores the urgent need for active surveillance and prompt infection-control measures. Several limitations of this study should nonetheless be acknowledged. First, it describes only two clinical isolates, which limits our ability to assess the population dynamics of resistance spread. Second, we did not perform comprehensive transcriptomic analysis to quantify *ftsI* expression changes under antimicrobial pressure. Finally, the fitness cost associated with the YRIK *ftsI* insertion remains to be evaluated in vivo.

## 4. Materials and Methods

### 4.1. Isolate Identification and Clinical Context

The two isolates were recovered at The Third Affiliated Hospital of Sun Yat-sen University on 3 September 2025 and 20 February 2026, respectively. The first clinical isolate, *Escherichia coli* ZSSY10862, was recovered from a cerebrospinal fluid (CSF) culture obtained from a 30-year-old male patient. Microorganisms isolated from various infectious sites comprised *Enterobacter cloacae*, *Klebsiella pneumoniae*, *Acinetobacter baumannii*, *Pseudomonas aeruginosa*, *Enterococcus faecium*, and *Candida auris* (resistance profiles are provided in Supplementary File S1), and the patient had prior antimicrobial exposure consisting of both empirical and targeted courses of piperacillin-tazobactam, meropenem, linezolid, ceftazidime-avibactam, tigecycline, vancomycin, cefoperazone-sulbactam, and posaconazole.

The other clinical isolate *Escherichia coli* ZSSY9436 was recovered from the sputum specimen obtained from a patient with polymicrobial pneumonia at the same hospital in February 2026. In addition to *Escherichia coli* ZSSY9436, *Pseudomonas aeruginosa*, *Acinetobacter baumannii* and *Candida auris* were also isolated from his sputum specimen (Supplementary File S1). This patient also had a history of intermittent exposure to various antimicrobial agents, including imipenem-cilastatin, cefoperazone-sulbactam, ceftazidime-avibactam, piperacillin-tazobactam, levofloxacin, colistin E (polymyxin E), tigecycline, doxycycline, and caspofungin.

Identification of both E. coli isolates was confirmed by matrix-assisted laser desorption ionization–time of flight mass spectrometry (MALDI-TOF MS) using a MicroFlex LT/SH system (Bruker Daltonics, Billerica, MA, USA).

### 4.2. Antimicrobial Susceptibility Testing and Carbapenemase Phenotyping

Antimicrobial susceptibility testing was performed by broth microdilution (BMD) according to Clinical and Laboratory Standards Institute (CLSI) guidelines. The antimicrobial susceptibility testing panel (bioMérieux, Marcy-l’Étoile, France) included cefoperazone-sulbactam, cefoxitin, cefuroxime, ceftazidime, ceftriaxone, cefepime, imipenem, ertapenem, piperacillin-tazobactam, levofloxacin, amikacin, tigecycline, trimethoprim-sulfamethoxazole, and amoxicillin-clavulanate. Susceptibility testing for minocycline, ceftazidime-avibactam, ampicillin, cefazolin, aztreonam, meropenem, and gentamicin was performed using the Kirby–Bauer disk diffusion method. Minimum inhibitory concentrations (MICs) of ATM-AVI were determined using a commercially available susceptibility panel (Wenzhou Kangtai, China) and confirmed by repeat testing. Interpretive criteria for ATM-AVI were applied according to EUCAST 2024 guidelines: susceptible (S) ≤8/4 µg/mL, resistant (R) >8/4 µg/mL. For other agents, CLSI M100-2025 breakpoints were applied.

Carbapenemase production was phenotypically detected using a combined-disk test with 3-aminophenylboronic acid (APB) and ethylenediaminetetraacetic acid disodium salt (Na_2_EDTA) (DL Biotech, Zhuhai, China). Four 10 μg imipenem disks were used: imipenem alone, imipenem plus APB, imipenem plus Na_2_EDTA, and imipenem plus both APB and Na_2_EDTA. Carbapenemase types were determined by comparing the inhibition zone diameters of the combined disks to those of imipenem alone, with a breakpoint of ≥4 mm [21].

### 4.3. DNA Extraction and Whole-Genome Sequencing

Genomic DNA was extracted from overnight bacterial cultures using genomic DNA extraction kits (Darui Biotech, Guangzhou, China) according to the manufacturer’s instructions. DNA integrity was evaluated by 1% agarose gel electrophoresis, DNA concentration was quantified using a Qubit 4.0 Fluorometer (Thermo Fisher Scientific, Carlsbad, CA, USA) and DNA purity was assessed using a NanoDrop One spectrophotometer (Thermo Fisher Scientific, Waltham, MA, USA).

For short-read sequencing, libraries were prepared using the ALFA-SEQ DNA Library Prep Kit (mCHIP, Guangzhou, China). Library concentration and fragment-size distribution were assessed using a Qubit 4.0 Fluorometer and the Qsep400 High-Throughput Nucleic Acid Protein Analysis System(BiOptic Inc. New Taipei City, Taiwan), respectively. Qualified libraries were sequenced on the Illumina NovaSeq 6000 (Illumina, San Diego, CA, USA) platform to generate 150 bp paired-end reads.

For long-read sequencing, libraries were prepared using the SQK-LSK109 kit (Oxford Nanopore Technologies, Oxford, UK) according to the manufacturer’s protocol. Library concentration was measured using a Qubit 4.0 Fluorometer, and average fragment size was estimated using an Agilent 4200 (Agilent, Santa Clara, CA, USA). Sequencing was performed on a Nanopore MinION (Oxford Nanopore Technologies, Oxford, UK) platform.

### 4.4. Genome Assembly, Annotation, and Characterisation

Hybrid genome assembly was performed using Illumina short-read and Nanopore long-read data with Unicycler v0.4.9 [22] under default parameters. Genome annotation was performed using Prokka 1.14.6 [23]. In silico multilocus sequence typing was conducted using mlst (https://github.com/tseemann/mlst, accessed on 28 February 2026) with the Achtman seven-gene scheme [24]. Antimicrobial resistance genes and resistance-associated determinants were identified using AMR FinderPlus 4.0.23 with database version 2025-07-16.1 [25]. The PBP3/*ftsI* alteration was initially identified from the AMRFinderPlus output as the resistance-associated determinant *ftsI*_I334IYRIK and was further confirmed by comparison of the PBP3/*ftsI* protein sequence with reference *E. coli* sequences. Plasmid features and predicted mobility were analyzed using MOB-suite (https://github.com/phac-nml/mob-suite accessed on 20 July 2026) [26]. Circular plots were generated using the online Proksee platform (https://proksee.ca) [27], with mobile genetic elements annotated using the integrated mobileOG-db module [28].

### 4.5. Phylogenetic and Comparative Genomic Analysis

Publicly available *E. coli* ST410 genomes were retrieved from NCBI GenBank, and 500 genomes most closely related to the study isolate *E. coli* ZSSY10862 were selected using Skani v0.3.1 [29]. Core-genome alignment and SNP detection were then performed using Parsnp 2.1.5 [30] against the reference genome *E. coli* JS316 (GenBank accession no. CP058618.1). Pairwise SNP distances among isolates were calculated from the Parsnp core SNP alignment using snp-dists (https://github.com/tseemann/snp-dists, accessed on 13 December 2025). For targeted comparative analysis, the clade containing *E. coli* ZSSY10862 and *E. coli* ZSSY9436 was extracted from the phylogenetic tree; this clade included isolates from diverse geographical origins and collection years. Antimicrobial resistance gene profiles and plasmid replicons among the selected comparative genomes were identified using abritAMR 1.0.20 [31] and ABRicate (https://github.com/tseemann/abricate, accessed on 13 December 2025) with the PlasmidFinder database, respectively. The resulting presence/absence matrices were visualized alongside the phylogenetic tree using Chiplot (https://www.chiplot.online/) [32].

## 5. Conclusions

We have characterized two clinical ATM-AVI-resistant *E. coli* ST410 isolates of international high-risk status that share a highly conserved chromosomal backbone, the same PBP3 alteration (*ftsI*_I334IYRIK), and *bla*_NDM-5_ carriage. These findings serve as a clinical alarm for infectious disease specialists and clinical microbiologists. As ATM-AVI becomes integrated into clinical practice, healthcare facilities must implement proactive surveillance for resistant clones. The window for preserving this valuable therapeutic option is narrowing. Our study underscores the urgent need for international collaborative surveillance, rigorous antimicrobial stewardship programs, and the development of next-generation β-lactamase inhibitor combinations active against PBP3-mutant strains.

## Figures and Tables

**Table 1:** Clinical characteristics and microbiological features of both isolates.

	*E. coli* ZSSY10862	*E. coli* ZSSY9436
Patient age/sex	30-year-old male	86-year-old male
Underlying disease	PCNS-DLBCL, hydrocephalus, VP shunt	Polymicrobial pneumonia and comorbidities
Specimen source	Cerebrospinal fluid	Sputum
Isolation date	3 September 2025	20 February 2026
Infection type	CNS infection/shunt-associated infection	Polymicrobial pneumonia
Condition upon discharge	Comatose, but vital signs stable and afebrile	Conscious, with stable vital signs and afebrile
Co-isolated pathogens from patient	*Enterobacter cloacae*, *Klebsiella pneumoniae*, *Acinetobacter baumannii*, *Pseudomonas aeruginosa*, *Enterococcus faecium*, and *Candida auris*	*Pseudomonas aeruginosa*, *Acinetobacter baumannii* and *Candida auris*
Prior antimicrobial exposure	piperacillin-tazobactam, meropenem, linezolid, ceftazidime-avibactam, tigecycline, vancomycin, cefoperazone-sulbactam, and posaconazole	imipenem-cilastatin, cefoperazone-sulbactam, ceftazidime-avibactam, piperacillin-tazobactam, levofloxacin, colistin E (polymyxin E), tigecycline, doxycycline, and caspofungin.
ATM-AVI MIC (µg/mL)	32	32
Ceftazidime-avibactam (CZA)	zone diameter: 10 mm	zone diameter: 10 mm
Carbapenems MICs (µg/mL)	≥16	≥16
Carbapenemase phenotype	MBL-positive	MBL-positive

**Table 2:** Genomic features of both isolates.

Feature	*E. coli* ZSSY10862	*E. coli* ZSSY9436
Genome size (bp)	5,158,521	5,196,370
Chromosome size	4,774,523 bp	4,873,256 bp
GC content (%)	50.5	50.4
MLST	ST410	ST410
rRNA/tRNA	22/96	22/93
Plasmid replicons	IncFIB(AP001918), IncFIC(FII), IncY, IncX1, IncX4, IncI2, ColRNAI, Col(BS512)	IncY, IncFIA, IncFIC, IncI2, IncX1, IncX4, ColRNAI-type, Col(MG828), Col(BS512)
AMR genes	*bla*_NDM-5_ (2 copies), *bla*_CTX-M-199_, *bla*_TEM-1_, *rmtB1*, *aadA2*, *dfrA12*, *sul1*, *sul2*, *tet(A)*, *aph(3’)-IIa*, *ble*	*bla*_NDM-5_, *bla*_CTX-M-137_, *bla*_CTX-M-14_, *bla*_TEM-1_, *bla*_OXA-10_, *rmtB1*, *aac(6’)-Ib3*, *aadA2*, *aadA1*, *dfrA12*, *dfrA14*, *sul1*, *tet(A)*, *arr-2*, *cmlA5*, *cmlA1*, *ble*
PBP3 alteration	*ftsI*_I334IYRIK	*ftsI*_I334IYRIK

**Table 3:** Predicted plasmid mobility of *bla*_NDM-5_-bearing elements in *E. coli* ZSSY10862 and *E. coli* ZSSY9436.

Sample ID/ Contig ID	Size	Rep Type(s)	Relaxase Type(s)	Mpf Type	Orit Type(s)	Mash Neighbor Identification	Predicted Mobility
*E. coli* ZSSY10862/2	136,125	IncFIB, IncFIC, rep_cluster_2244	MOBF, MOBP	MPF_F	MOBF	*Escherichia* *coli*	conjugative
*E. coli* ZSSY9436/5	53,604	IncX1	MOBQ	-	-	*Klebsiella pneumoniae*	-
*E. coli* ZSSY 9436/3	66,551	IncFIA, IncFIC	-	MPF_F	MOBF	*Klebsiella pneumoniae*	-
*E. coli* ZSSY9436/3+5(hypothesized)	120,155	IncFIA, IncFIC, IncX1	MOBQ	MPF_F	MOBF	*Klebsiella pneumoniae*	conjugative

## Data Availability

The assembled genomes generated in this study have been deposited in the Genome Sequence Archive at the National Genomics Data Center (NGDC), China National Center for Bioinformation, under accession number PRJCA066460.

## Supplementary Materials

The following supporting information can be downloaded at: https://www.mdpi.com/article/10.3390/antibiotics15080750/s1, File S1: co-isolated organisms.

## Abbreviations

The following abbreviations are used in this manuscript:

ATM-AVI	Aztreonam–avibactam
CRE	Carbapenem-resistant Enterobacterales
MBLs	Metallo-β-lactamases
NDM	New Delhi metallo-β-lactamase
ESBLs	Extended-spectrum beta-lactamases
PBP3	Penicillin-binding protein 3
*E. coli*	*Escherichia coli*
CSF	Cerebrospinal fluid
PCNS-DLBCL	Primary central nervous system diffuse large B-cell lymphoma
SNP	Single-nucleotide polymorphism
ECDC	European Centre for Disease Prevention and Control
