# Supplementary material for: Clinical Emergence and Genomic Characterization of Aztreonam–Avibactam-Resistant Escherichia coli ST410 Isolates in China
Source: Antibiotics (Basel). 2026 Aug 3;15(8):750. doi: 10.3390/antibiotics15080750 (PMC13509860; doi:10.3390/antibiotics15080750)
Supplement: Supplementary file 1 [file antibiotics-15-00750-s001.zip › antibiotics-4423519-supplementary.pdf]

Supplementary File S1: co-isolated organisms.

To provide a comprehensive overview of the microbiological context, we have compiled the resistance profiles of all co-isolated organisms and have included this information as a supplementary file.

Patient 1 (*E. coli* ZSSY10862):

(1) *Enterobacter cloacae* was isolated from sputum, which was multidrug-resistant bacteria (MDR) and resistant to cephalosporins, fluoroquinolones, and aminoglycosides, but also remained susceptible to common beta-lactam/beta-lactamase inhibitor combinations and carbapenems.

(2) *Klebsiella pneumoniae* was isolated from sputum, which was multidrug-resistant bacteria (MDR), produced Extended-spectrum Beta-lactamase (ESBL) and was resistant to cephalosporins and common inhibitor combinations such as ampicillin/sulbactam and piperacillin/tazobactam, but also remained susceptible to carbapenems.

(3) *Acinetobacter baumannii* was isolated from bronchoalveolar lavage fluid (BALF), which was an carbapenem-resistant Acinetobacter baumannii (CRAB) and susceptible only to colistin and sulbactam/durlobactam.

(4) *Pseudomonas aeruginosa* was isolated from bronchoalveolar lavage fluid (BALF), which was an carbapenem-resistant *Pseudomonas aeruginosa* (CRPA) .

(5) *Enterococcus faecium* was isolated from the cerebrospinal fluid, which was sensitive to common drugs except erythromycin.

(6) *Candida auris* was isolated from from bronchoalveolar lavage fluid (BALF) with an elevated MICs for fluconazole (MIC, 256mg/L), amphotericin B (MIC, 2mg/L), and 5-fluorocytosine (MIC, 32mg/L) according to the tentative resistance breakpoints determined by the U.S. CDC.

Patient 2 (*E. coli* ZSSY9436):

(1) Pseudomonas aeruginosa was isolated from sputum, which was an carbapenem-resistant *Pseudomonas aeruginosa* (CRPA) and also resistant to fluoroquinolones.

(2) Acinetobacter baumannii was isolated from bronchoalveolar lavage fluid (BALF), which was an carbapenem-resistant Acinetobacter baumannii (CRAB) and were susceptible only to colistin and sulbactam/durlobactam.

(3) Candida auris was isolated from sputum and bronchoalveolar lavage fluid (BALF), which was relatively low MICs for fluconazole (MIC≤2 mg/L), amphotericin B (MIC≤0.5 mg/L), and 5-fluorocytosine (MIC≤4 mg/L) according to the tentative resistance breakpoints determined by the U.S. CDC.
